# Development of a theory of implementation and integration: Normalization Process Theory

**DOI:** 10.1186/1748-5908-4-29

**Published:** 2009-05-21

**Authors:** Carl R May, Frances Mair, Tracy Finch, Anne MacFarlane, Christopher Dowrick, Shaun Treweek, Tim Rapley, Luciana Ballini, Bie Nio Ong, Anne Rogers, Elizabeth Murray, Glyn Elwyn, France Légaré, Jane Gunn, Victor M Montori

**Affiliations:** 1 Institute of Health and Society, Newcastle University, Newcastle, UK; 2 Division of General Practice, Glasgow University, Glasgow, UK; 3 Department of General Practice, National University of Ireland, Galway, Ireland; 4 School of Population and Behavioural Sciences, University of Liverpool, Liverpool, UK; 5 Centre for Primary Care and Population Research, University of Dundee, Dundee, UK; 6 Agenzia Sanitaria e Sociale Regionale, Bologna, Italy; 7 Arthritis Research Campaign National Primary Care Centre, Keele University, Keele, UK; 8 National Primary Care Research and Development Centre, University of Manchester, Manchester, UK; 9 Department of Primary Care, University College London, London, UK; 10 Department of Primary Care and Public Health, School of Medicine, Cardiff University, Cardiff, UK; 11 Department of Family Medicine, Université Laval, Québec, Québec, Canada; 12 Department of General Practice, University of Melbourne, Melbourne, Australia; 13 Knowledge and Encounter Research Unit, Mayo Clinic, Rochester MN, USA

## Abstract

**Background:**

Theories are important tools in the social and natural sciences. The methods by which they are derived are rarely described and discussed. Normalization Process Theory explains how new technologies, ways of acting, and ways of working become routinely embedded in everyday practice, and has applications in the study of implementation processes. This paper describes the process by which it was built.

**Methods:**

Between 1998 and 2008, we developed a theory. We derived a set of empirical generalizations from analysis of data collected in qualitative studies of healthcare work and organization. We developed an applied theoretical model through analysis of empirical generalizations. Finally, we built a formal theory through a process of extension and implication analysis of the applied theoretical model.

**Results:**

Each phase of theory development showed that the constructs of the theory did not conflict with each other, had explanatory power, and possessed sufficient robustness for formal testing. As the theory developed, its scope expanded from a set of observed regularities in data with procedural explanations, to an applied theoretical model, to a formal middle-range theory.

**Conclusion:**

Normalization Process Theory has been developed through procedures that were properly sceptical and critical, and which were opened to review at each stage of development. The theory has been shown to merit formal testing.

## Background

Theories are important for the social and natural sciences because they make possible robust explanations of previously or currently observed phenomena, and because they are points of departure for forecasts about future phenomena. There are now a number of important and useful theories of individual and group behaviour that can be applied to understanding implementation problems [1-4], and this paper described the processes by which one of them – Normalization Process Theory (or NPT) [5-7] – was developed between 2000 and 2009. The paper is an account of the development of NPT. The objective of this paper is to describe the procedures by which the theory was built. We show how these procedures led to a set of propositions that possessed sufficient face validity and conceptual robustness to warrant formal testing. That work is currently underway and its results will be reported in due course

### What is NPT?

NPT provides a set of sociological tools to understand and explain the social processes through which new or modified practices of thinking, enacting, and organizing work are operationalized in healthcare and other institutional settings. In particular, the theory is concerned with three core problems:

1. Implementation, by which we mean the social organization of bringing a practice or practices into action.

2. Embedding, by which we mean the processes through which a practice or practices become, (or do not become), routinely incorporated in everyday work of individuals and groups.

3. Integration, by which we mean the processes by which a practice or practices are reproduced and sustained among the social matrices of an organization or institution.

The theory is described in detail elsewhere [5-7]. In summary, however, it is postulated that:

1. Practices become routinely embedded – or normalized – in social contexts as the result of people working, individually and collectively, to enact them.

2. The work of enacting a practice is promoted or inhibited through the operation of generative mechanisms (coherence, cognitive participation, collective action, reflexive monitoring) through which human agency is expressed.

3. The production and reproduction of a practice requires continuous investment by agents in ensembles of action that are carried forward in time and space.

The starting point of the theory is that to understand the embedding of a practice we must look at what people actually do and how they work. It is a theory of action. This distinguishes it from theories of the cultural transmission of innovations (such as Diffusion of Innovations Theory [8,9]) that seek to explain how innovations spread; theories of collective and individual learning and expertise [10] that seek to explain how innovations are internalized; and theories of the relationships between individual attitudes and intentions and behavioural outcomes [11]. The explanatory focus of the theory, and its emphasis on human agency, sets it apart from sociological theories of actor-networks [12] that take ethnographic case studies at their primary method [13], wrongly attribute agency to things as well as people, and explicitly reject explanation in favour of description [11]. In contrast to the latter, the aim here has been to build a set of sociological tools to investigate social shaping as action, and to do this in a form that permits structured comparative inquiry prospectively using a variety of methods.

### Why was NPT developed?

The contingent and complex relational processes involved in theory building are not the focus of this paper. But it is important to address the question of why NPT came about. The key problem it was developed to address was the observed difficulty of implementing and integrating new treatment modalities and ways of organizing care in health service settings. Specifically, some of us sought to address a perceived gap in the tools available to explain the failure of apparently widely adopted and diffused telemedicine systems to become routinely incorporated in clinical settings, even in circumstances where professionals were favourably disposed to them, and where significant material political support was committed to them [14]. Reviews of relevant theory published at that time [1-4] explicated these explanatory gaps. Indeed, these reviews seem to have been inspired by the recognition of the lack of strong theoretical basis for the planning and evaluating of implementation programmes. They reflect calls for the use of theories to generate testable hypotheses linking tailored strategies with factors that promote or inhibit implementation, and various attempts to identify theories or constructs within theories best suited for successful implementation [15]. The development of NPT is one response to these calls, and has the added value of being derived from empirical generalizations developed within studies of implementation and integration processes, rather than being derived from plausibly useful constructs embedded in other theories.

Procedural accounts of theory-building risk imposing an artificial order on processes that are highly contingent. Such processes are, in practice, very difficult to map. In this instance, the social relations and processes that have led to the development of NPT are complex, as are the research and policy problems and networks in which they are located. However, we can say that the processes of theoretical development described in this paper were opportunistic and organic in the beginning. However, after a formal Normalization Process Model [7] was developed and presented at seminars and conferences during 2005 and 2006, a multi-disciplinary group of researchers formed around the model and began to refine and develop it – and, most importantly, to apply it to specific research problems. After this group formed, theoretical development was undertaken more deliberately and strategically, with formal meetings in 2007 and 2008. After 2008, development of NPT was support by funding for meetings of a Peer Learning Set from the UK National Institute of Health Research, and by a 'follow-on' grant from the UK Economic and Social Research Council.

## Methods

### Phase one: Developing empirical generalizations

Between 2001 and 2004, data collected in qualitative studies of healthcare work and organization was subjected to secondary analyses, and sets of empirical generalizations were derived from these. These related to four domains of research: the normalization of telemedicine systems [16,17]; professional-patient interaction and the organization of healthcare work in chronic illness [18,19]; and the social production and operationalization of evidence in the clinical encounter [20]. These treated 'normalization' as the endpoint of an implementation process in which some new technology came to be routinely employed in service. The comparative synthetic methods used to generate empirical generalizations about telemedicine implementation processes were then used to perform a similar analysis on data collected in the other studies. The methods by which secondary analyses were carried out have been described in detail elsewhere [7].

The empirical generalizations produced by these processes were general conclusions about regularities in the data, and were framed as formal propositions. They are given in Appendix 1. They did not in themselves make a theory because they were specific to particular contexts (*i.e.*, although they were generalizations, they were not necessarily generalizable), and were not linked together by some account of causal relations, generative mechanisms, or organizing principles. In other words, they were observational rather than explanatory.

### Phase two: Building an applied theoretical model

Between 2003 and 2007, using grounded theory-building techniques [21,22], an applied theoretical model of normalization processes – the Normalization Process Model, or NPM, [7] – was derived from earlier empirical generalizations across all four domains of study. This was framed as a set of analytic propositions (see Appendix 2) that were supported by rigorous data analysis. This aimed to develop what Stinchcombe [23] has called an applied theoretical model of the factors that promote or inhibit the work of routine embedding of some new health technology in practice. These were first subject to critical review from a large group of researchers to whom manuscripts of different iterations of the model were informally circulated, and discussed at a series of seminars.

The purpose of the NPM was to identify and explain those factors that promoted or inhibited collective action that led to the routine embedding of complex healthcare interventions in service settings. There were four of these (interactional workability, relational integration, skill-set workability, and contextual integration) and we defined these as the constructs of the NPM. At this stage, the NPM synthesized empirical generalizations from groups of related studies, and producing taxonomies, maps of relations between concepts, and generalizations [24]. These were linked together by sociological explanations of the relations between its constructs, their dimensions, and components. Taken together these set the scene for possible empirical verification.

### Refining and testing the NPM

As an applied theoretical model, the NPM was restricted to a specific field of activity: the operationalization of complex healthcare interventions [7]. To develop it further we sought to define and stabilise the way that we conceptualized theory itself. We assigned to theory three kinds of work [6]:

1. Accurate description: A theory must provide a taxonomy or set of definitions that enable the identification, differentiation, and codification of the qualities and properties of cases and classes of phenomena.

2. Systematic explanation: A theory must provide an explanation of the form and significance of the causal and relational mechanisms at work in cases or classes of the phenomena defined by the theory, and should propose their relation to other phenomena.

3. Knowledge claims: A theory must lead to knowledge claims. These may take the form of abstract explanations, analytic propositions, or experimental hypotheses.

Further development of the NPM involved applying it empirically, in a process of 'road testing' the theoretical model [25]. An important critique of theory building is that it is sometimes precipitate, proceeding before the generalizability of the phenomena it is concerned with is properly established [26]. A second critique is that theory-builders focus too early on the problem of defining and measuring variables supposed to be relevant, without sufficient consideration of the coherence and robustness of basic concepts and constructs of the theory itself [27]. 'Road testing' the NPM enabled us to work through these problems and provided a context in which to make rational decisions about face validity, and to ask whether the NPM merited formal testing. This consisted of two main pieces of work, quantitative data analysis and research synthesis.

### Qualitative data analysis

We integrated the NPM in qualitative data analysis in three large studies (of the implementation of e-health technologies [28], the integration of telecare systems [29,30], and the operationalization of a large randomized controlled trial). As we did this, we sceptically sought evidence for the adequacy of the NPM to perform the three functions of theory that we had previously claimed for it – to define phenomena, explain mechanisms, and form knowledge claims. It is important to be clear that this was not formal testing, because we did not at this stage seek to falsify the NPM. Instead, we practically tested its usefulness as an analytic tool.

### Research synthesis

Elwyn *et al*. [31] undertook a parallel critical analysis of the NPM by applying it to the problems of operationalizing shared decision-making tools in medical consultations. Participants in that process mapped the constructs of the NPM against data from evaluation and other literature, including primary studies and systematic reviews, and produced a set of attributions about the conclusions of these studies. The NPM was then applied to these attributions to determine whether it usefully explained them. Elwyn *et al*. [31] concluded that the NPM offered stable explanations of the collective work involved in shared decision-making processes and operationalizing decision-making tools.

By the end of 2006, the NPM in its published form [7] was sufficient as a set of conceptual tools to analyse specific processes, and it has been successfully applied to this purpose [32-36]. 'Road testing' showed that it had utility in explaining factors that promoted and inhibited collective action in operationalizing practices. It did not, however, explain how practices were formed in ways that held together, how actors were enrolled into them, or how they were appraised. These were three domains in which NPM could usefully be expanded. This recognition informed the next stage of theory building.

### Phase three: Making a formal theory

After 2006, we worked to solve these problems. Between 2006 and 2009, the applied theoretical model of the NPM was extended, new constructs defined, and generative mechanisms defined, so that it formed a formal middle-range theory – NPT.

The production of a formal theory is a quite different enterprise than the work that goes into the identification of empirical generalizations or applied explanations. The goal of theory-building at this level is to isolate the generic properties of phenomena and understand their operation [37]. To do this, we had to reformulate the healthcare-specific constructs of the NPM as generic or abstract propositions, and then to extend the theory by writing three constructs that related to domains we had previously established were absent. We called these coherence, cognitive participation, and reflexive monitoring. Although at this stage we still regarded our work as framing an extended NPM, we had embarked on a process that would lead to a generalizable, middle-range, formal theory:

1. We had defined NPM constructs as factors that promoted or inhibited collective action leading to the routine embedding of some intervention. We used additional analyses to identify macro-level analogues of the constructs of the model [30,38]. These took the form shown in Appendix 3. We then constructed full definitions of the macro-level analogues of the NPM constructs and tested them against already collected data.

2. We operationalized macro-level constructs in a way that mapped on to the existing constructs of the NPM (see Appendix 4). For example, we construed collective action as a macro-level construct (with micro-level constructs of interactional workability, relational integration, skill-set workability, and contextual integration).

3. As we worked through macro-level constructs, we also began to use a much more structured model of theory-building in which generative mechanisms and relations required definition [39,40]. In this context, we shifted attention to coherence work not as a macro-level abstraction of contextual integration, but rather as a generative mechanism through which an intervention was subjected to sense-making procedures by its users.

4. We drew maps of the processes with which we were concerned. This method for identifying the constituents of conceptual models is called analytical theorizing by Turner [37]. This led to a map of the expanded NPM at work. We then followed Lieberson and Lynn [40] in reframing macro-level constructs derived from the NPM as descriptors of 'generative principles'.

The extended NPM that was derived from this work now had a general character, and the generative mechanisms and components to which it referred were not exclusive to complex interventions or even healthcare. They referred instead to generic properties of implementation processes and offered an explanation of them without reference to specific social contexts. We therefore presented it as a general, and generalizable, middle-range theory, NPT [5,41]), that seeks to explain the processes of implementation, embedding, and integration of material practices in formally defined contexts, relates these processes to causal social mechanisms [42], identifies components of those mechanisms, and defines the investments that are required to energize them. The mechanisms of the NPT are described in detail elsewhere [5], but synopses are provided in Appendix 4 and Appendix 5.

### Road testing the NPT

Just as development of the NPM involved a process of 'road testing' to decide whether it was sufficiently plausible and robust to merit formal testing, so did the NPT. We accomplished this using multiple methods. It is important to emphasise that the purpose of this work was not to formally test the theory, but rather to demonstrate that it was fit to be tested:

1. Assessing the stability of NPT constructs: Researchers working in very different contexts and on very different studies (including studies of e-health implementation and reconfiguration of primary care mental health services in the State of Victoria, Australia) worked with the constructs of the NPT to develop analyses of implementation and embedding processes [43,44]. The criteria for stability were that the generic constructs could be translated into specific contexts without the addition of ad hoc conditions, and that sceptical researchers were able to use them in practice with minimal support.

2. Critical comparison of NPM and NPT constructs: A key question was whether or not expanding the scope of normalization process analysis to the higher-level constructs of the NPT has practical value. In other words, we wanted to be clear that there was an advantage to using the NPT. To this end, we coded two sets of data (interview transcripts from a study of e-health implementation processes, and qualitative data collected in systematic review of e-health implementation studies) using both the NPM and NPT [43].

To summarise, 'road testing' NPT required that we establish that its constructs actually defined mechanisms, components, and investments that could all be prospectively revealed by empirical research, and that these could be characterised in a stable way. We then had to demonstrate that these constructs could be operationalized in a way that conferred an analytic advantage. We sought confidence that NPT covered the ground we claimed for it, and that propositions could be derived from it that could effectively test the data and explain phenomena. This process was important because it paralleled the final revisions of the NPT as subsequently accepted for publication.

### Relationship between the NPM and NPT

The formal theory (NPT) does not conflict with the applied model (NPM) from which it was drawn. In fact, it extends it. The constructs of the NPM are central to the formal theory and constitute its collective action component. The NPM is unchanged by this, and researchers can continue to successfully use the NPM in settings where only those factors that promoted or inhibited collective action are at issue [32-35,45,46]. The NPT, however, extends the applied theoretical model to include the ways by which actors make sense of a set of practices (coherence), the means by which they participate in them (cognitive participation), and the forms of appraisal that they apply (reflexive monitoring)

### NPT is a middle-range theory

Although it has been developed through a series of multi-disciplinary collaborations, NPT is a sociological theory in that it takes as its focus the contribution of social action to implementation, embedding, and integration. It is also a middle-range theory [47,48]. Following Merton [49]), we use this term to mean the following: the theory is 'sufficiently abstract to be applied to different spheres of social behaviour and structure' but does not offer a set of general laws about behaviour and structure at a societal level; the scope of the theory is defined by a limited set of assumptions from which can be derived hypotheses that may be confirmed or disconfirmed by empirical investigation; the limited scope of the theory leads to the 'specification of ignorance'. That is, the limits of explanation within the frame of the theory are established, and it does not 'pretend to knowledge where it is in fact absent'.

Specifying the range of the theory is important. Recent debates about theory in the social sciences [13,50,51] have emphasised the search for 'medium-scope patterns and mechanisms [that] distinguish between a complex social reality and an intentionally simplified analytical model of this reality' [50]. The limited scope, conceptual range, and claims of middle-range theories are important because they are what make them practically workable in analysing practice.

## Results

### The changing scope of the theory

This paper has described the procedures by which NPT was developed. The development of a set of explanatory ideas around normalization has shifted from an initial set of empirical generalizations presented as synthetic propositions or assertions [17], to a robust conceptual model that presented generalizable propositions [7], and finally to a middle-range theory that offers a set of mechanism-based explanations for processes of implementation, embedding, and integration [5]. This has involved a steady shift away from context dependent statements. The processes of theory development described here have included changes in scope, as well as method. Simply accounting for this is unusual, but is a necessary precondition for research that subjects the NPT to formal and definitive tests. It is important to show that the theory has been derived through processes that have involved the application of rigorous methods, that these methods have been applied in a properly sceptical way, and that the outcomes of their application have been critically assessed.

### The importance of transparency

Despite different streams of writing about theory development in the social sciences – for example, writing around the construction of grounded theory [21,22,51-54], and about the development of formal sociological theory [23,54,55] – we actually have few factual accounts of the development of theories themselves. When they exist, these often take the form of personal histories [56], or accounts of particular social networks [57]. So, although there are many papers that seek to present some new theory, we can often discover little about where they come from or about the methods by which they were derived. Unless there is already a large body of literature that presents studies that have interrogated or tested a particular theory in play, we are then stuck with the problem of how to evaluate its relation to the phenomena that it seeks to explain. Such theories sometimes seem to spring fully-formed from critiques of the literature, or by assertions about prior theories. We have sought to avoid this problem.

## Conclusion

The process of theory-building described in this paper has led from secondary analysis of qualitative data through to the development of a set of generic theoretical propositions that can be employed to explain implementation and integration. Our aim in the work described in this paper has been to develop an explanatory model that can underpin structured, prospective studies that have both practical and policy relevance, and which are genuinely open to interdisciplinary inquiry. This theory-building process has been a highly collaborative one, in which many people have made very important contributions to the development of theoretical explanation. None of those who participated in the first stage of theory-building described earlier in this paper recognised that they were involved in a process that would, subsequently, need to be accounted for in a paper such as this. At this stage, it is therefore important to describe the procedures by which the enterprise of theory building has been accomplished. The theory itself is described in detail elsewhere [5], and accounts of research that tests the theory, for good or ill, are forthcoming.

## Competing interests

The authors declare that they have no competing interests.

## Authors' contributions

Authorship is ordered according to the time-point at which contributors joined in the work of theory development described in this paper, and their contributions are attributed on the following basis. The programme of theory-building was led by CRM. He also drafted this manuscript (with substantial assistance from AMacF). Phase one included CRM, FM, TF and AMacF; Phase two included CRM, FM, TF, CFD, AMacF, ST, TR, BNO, AR, EM, GE, and FL; in Phase three, formal theory development was done by CRM and TF. Other contributions to work in this phase were made by FL, FM, EM, JG, and VM. All authors have contributed practically and intellectually to the work that led to this paper and have commented and agreed on the manuscript.

## Appendix

### Appendix 1 – Empirical generalizations on normalization processes for telemedicine (2003) [17]

P_1 _Implementation of telemedicine services depends on a positive link with a (local or national) policy level sponsor, so that telemedicine is defined as an appropriate means of delivering care, and appropriate infrastructures are developed.

P_2 _Adoption of telemedicine systems in service depends on successful integration at the level of structural legitimation so that it is supported as, and thus practically incorporated into, health care delivery through the development of organizational structures.

P_3 _Translation of telemedicine technologies into clinical practice depends on the enrolment of heterogeneous actors into relatively cohesive, co-operative groups, in which functional identities are negotiated and established a priori and powers relatively well defined.

P_4 _Stabilization of telemedicine systems in practice depends on integration at the level of professional knowledge and practice, where clinicians are able to accommodate telemedicine in their clinical activities through the development of new procedures and protocols.

P_5 _The normalisation of telemedicine as a means of health care delivery (in whatever setting, and at whatever level of healthcare provision) is conditional on P_1 _+ P_2 _+ P_3 _+ P_4_.

### Appendix 2 – Propositions of the Normalization Process Model (2006) [7]

1. A complex intervention is disposed to normalization if it confers an interactional advantage in flexibly accomplishing congruence and disposal;

2. A complex intervention is disposed to normalization if it equals or improves accountability and confidence within networks;

3. A complex intervention is disposed to normalization if it is calibrated to an agreed skill-set at a recognizable location in the division of labour;

4. A complex intervention is disposed to normalization if it confers an advantage on an organization in flexibly executing and realizing work.

### Appendix 3 – Intermediate development of the theory – macro to micro links between constructs [39]

#### Domain of work (macro level)

(Defined as generative mechanisms in Normalization Process Theory)

**Coherence: **Work that defines and organizes the objects of a practice.

**Cognitive participation: **Work that defines and organizes the enrolment of participants in a practice.

**Collective action: **Work that defines and organizes the enacting of a practice.

**Reflexive monitoring: **Work that defines and organizes the knowledge upon which appraisal of a practice is founded.

#### Everyday practices (micro level)

(Defined as constructs of the Normalization Process Model)

Practices that ensure **contextual integration **with healthcare systems and services

Practices that are defined by their **skill-set workability **within formal and informal divisions of healthcare labor.

Practices that are defined by their **interactional workability **within a set of everyday social relations.

Practices that ensure **relational integration **of knowledge and practice in a network of actors

### Appendix 4 – General Propositions of Normalization Process Theory (2009) [5]

1. Material practices become routinely embedded in social contexts as the result of people working, individually and collectively, to implement them. From this follows specific propositions that assert that define a mechanism (*i.e.*, embedding is dependent on socially patterned implementation work).

2. The work of implementation is operationalized through four generative mechanisms (coherence, cognitive participation, collective action, reflexive monitoring). From this follows specific propositions that define components of a mechanism (*i.e.*, those factors that shape socially patterned implementation work).

3. The production and reproduction of a material practice requires continuous investment by agents in ensembles of action that carry forward in time and space. From this follows specific propositions that define actors' investments in a mechanism (*i.e.*, how the mechanism is energized).

### Appendix 5 – Specific propositions of Normalization Process Theory (2009) [5]

#### Coherence

Routine embedding is dependent on work that defines and organizes a practice as a cognitive and behavioural ensemble.

Embedding work is shaped by factors that promote or inhibit actors' apprehension of a practice as meaningful.

The production and reproduction of coherence in a practice requires that actors collectively invest meaning in it.

#### Cognitive participation

Routine embedding is dependent on work that defines and organizes the actors implicated in a practice.

Embedding work is shaped by factors that promote or inhibit actors' participation.

The production and reproduction of a practice requires that actors collectively invest commitment in it.

#### Collective action

Routine embedding is dependent on work that defines and operationalizes a practice.

Embedding work is shaped by factors that promote or inhibit actors' enacting it.

The production and reproduction of a practice requires that actors collectively invest effort in it.

#### Reflexive monitoring

Routine embedding is dependent on work that defines and organizes the everyday understanding of a practice.

Embedding work is shaped by factors that promote or inhibit appraisal.

The production and reproduction of a practice requires that actors collectively invest in its understanding.
